# Sirt5 Attenuates Cisplatin-Induced Acute Kidney Injury through Regulation of Nrf2/HO-1 and Bcl-2

**DOI:** 10.1155/2019/4745132

**Published:** 2019-11-14

**Authors:** Wei Li, Yuanyuan Yang, You Li, Yueyue Zhao, Hong Jiang

**Affiliations:** Department of Pediatrics, The First Affiliated Hospital of China Medical University, Shenyang, China

## Abstract

Cisplatin- (CDDP) induced acute kidney injury (AKI) limits the clinical use of cisplatin. Several sirtuin (SIRT) family proteins are involved in AKI, while the roles of Sirt5 in cisplatin-induced AKI remain unknown. In the present study, we characterized the role and mechanism of Sirt5 in cisplatin-induced apoptosis using the human kidney 2 (HK-2) cell line. CDDP treatment decreased Sirt5 expression of HK-2 cells in a dose-dependent manner. In addition, Sirt5 overexpression enhanced the metabolic activity in CDDP-treated HK-2 cells while Sirt5 siRNA attenuated it. Forced expression of Sirt5 inhibited CDDP-induced apoptosis while Sirt5 siRNA showed the opposite effects. Accordingly, Sirt5 overexpression inhibited the level of caspase 3 cleavage and cytochrome c levels. Furthermore, we found that Sirt5 increased mitochondrial membrane potentials and ameliorated intracellular ROS production. Mitotracker Red staining indicated that Sirt5 overexpression was able to maintain the mitochondrial density during CDDP treatment. We also investigated possible downstream targets of Sirt5 and found that Sirt5 increased Nrf2, HO-1, and Bcl-2 while it decreased Bax protein expression. Sirt5 siRNA showed the opposite effect on these proteins. The levels of Nrf2, HO-1, and Bcl-2 proteins in HK-2 cells were also decreased after CDDP treatment. Moreover, Nrf2 and Bcl-2 siRNA partly abolished the protecting effect of Sirt5 on CDDP-induced apoptosis and cytochrome c release. Catalase inhibitor 3-AT also abolished the cytoprotective effect of Sirt5. Together, the results demonstrated that Sirt5 attenuated cisplatin-induced apoptosis and mitochondrial injury in human kidney HK-2 cells, possibly through the regulation of Nrf2/HO-1 and Bcl-2.

## 1. Introduction

Cisplatin is one of most commonly used chemotherapeutic drugs in the treatment of solid tumors including liver [1], lung [2], breast [3], cervical [4], ovarian [5], and testis [6] cancers. Although cisplatin has been shown to be one of the most effective anticancer drugs, its use in clinical application is limited because of its side effects in normal tissues [7–9]. The major consequences during cisplatin treatment are nephrotoxicity, ototoxicity, and neurotoxicity. Cisplatin tends to accumulate in the kidneys more than in other organs. Cisplatin-induced acute kidney injury (AKI) has therefore been recognized as a major concern and limits its use in cancer treatment.

Sirtuins (SIRTs) are a protein family of nicotinamide adenine dinucleotide- (NAD^+^-) dependent histone deacetylases, which are involved in a series of biological processes including DNA damage repair, aging, oxidative stress, and inflammation response [10–14]. There are seven members (SIRT1-7) in the SIRTs family, which display different intracellular locations, enzymatic activities, and biological functions [15, 16]. Several reports have shown that SIRTs are involved in cisplatin-induced AKI. A previous study indicated that SIRT7 knockout mice showed a protective effect against cisplatin-induced AKI through regulating the NF-*κ*B signaling and TNF*α* expression. Another report showed that SIRT1 was decreased by cisplatin treatment when compared with control buffer treatment. However, the effect of Sirt5 on cisplatin-induced AKI is unknown.

Kidney is second only to the heart in mitochondrial count and oxygen consumption. Therefore, mitochondrial homeostasis is pivotal to normal kidney function. Dysregulation of mitochondrial biogenesis is involved in many renal diseases including AKI, and mitochondrial dynamics is perturbed in nephrotoxic and septic AKI [17, 18]. Increased mitochondrial ROS formation has been observed in both chronic and acute renal diseases [19]. In addition, convincing evidence has shown mitochondrial-related intrinsic apoptosis in AKI [20]. The study of mitochondrial dysfunction has therefore emerged as an exciting new area to identify therapies for AKI [21].

In this study, we characterized the biological effects and the potential mechanisms of action of Sirt5 in cisplatin-induced AKI using HK-2 human kidney 2 (HK-2) cell line which is derived from proximal tubule epithelium of the normal human kidney.

## 2. Materials and Methods

### 2.1. Cell Culture

HK-2 cells were purchased from American Type Culture Collection (ATCC, Manassas, VA, USA) and cultured in keratinocyte serum-free medium (K-SFM, Gibco, Waltham, MA, USA) with 0.05 mg/mL bovine pituitary extract (BPE) and 5 ng/mL human recombinant epidermal growth factor (EGF). Cells were maintained in a humidified atmosphere at 37°C with 5% CO_2_ and subcultured every 3 days. Catalase inhibitor (3-amino-1,2,4-triazole, 3-AT) was purchased from Santa Cruz (USA).

### 2.2. Sirt5 Plasmid Transfection

HK-2 cells were seeded in 6-well plates at a density of 5 × 10^5^ cells per well at 37°C in a 5% CO_2_ incubator until they reached 60–80% confluence. pCMV6-Sirt5 plasmid and the matched empty plasmid were transfected into the cells using Lipofectamine 3000 (Invitrogen, Carlsbad, CA, USA) according to the manufacturer's instructions.

### 2.3. Small Interfering siRNA Transfection

HK-2 cells (5 × 10^5^ cells per well) were incubated in K-SFM medium at 37°C in a 5% CO_2_ incubator until they reached 60–80% confluence. The Sirt5 siRNA (Dharmacon, Lafayette, IN, USA) and nontargeting siRNA were transfected into HK-2 cells using DharmaFECT 1 Transfection Reagent according to the manufacturer's instructions.

### 2.4. Real-Time Fluorescence Quantitative PCR

Total RNA from HK-2 cells was extracted using the TRIzol reagent (Thermo Fisher Scientific, Waltham, MA, USA) and was quantified using Nanodrop2000c (Thermo Fisher Scientific). cDNA synthesis was performed using a Prime Script RT Master Mix (TaKaRa) at 85°C for 2 min and 37°C for 30 min. Quantitative real-time PCR was performed using SYBR Green Master mix (Applied Biosystems, MA, USA) at 50°C for 2 min, 95°C for 2 min, and 45 cycles of 95°C for 15 sec and 60°C for 40 sec. ABI 7500 Thermal Cycler (Applied Biosystems) was used for quantitative real-time PCR. The primers used in this study were listed as follows: Sirt5 forward 5′-AGTACCAGACTGCCCTGA-3′; Sirt5 reverse 5′-CACTCCCACTGTCCTTTC-3′. GAPDH forward 5′-ATGATGACATCAAGAAGGTGG-3′; GAPDH reverse 5′-TTGTCATACCAGGAAATGAGC-3′. The relative levels of gene expression were represented as ΔCt = Ct gene–Ct reference, and the fold change of gene expression was calculated by the 2^−ΔΔCt^ method.

### 2.5. Western Blot

Whole cell protein was collected using ice-cold lysis buffer with protease inhibitor cocktail and phosphatase inhibitor cocktail (Thermo Fisher Scientific). The samples were centrifuged at 10000 rpm at 4°C for 10 minutes, and the supernatant lysate was collected. The protein concentration was estimated using the Pierce BCA protein assay reagent kit (Thermo Fisher Scientific). A protein sample is mixed with the 4x *Laemmli sample buffer* (Bio-Rad) and heated in boiling water for 5 minutes. A total of 40 *μ*g of protein was separated using SDS-PAGE and then transferred onto a PVDF membrane. The membrane was blocked in 5% BSA diluted with TBST buffer. The membrane was incubated with the following primary antibodies overnight at 4°C: Sirt5 (1 : 1000, cell signal technology, USA); Nrf2 (1 : 1000, Santa Cruze, USA); Drp1 and p-Drp1 (Ser616); HO-1, Bcl-2, cytochrome c, caspase 3, cleaved caspase 3, and Bax (1 : 1000, cell signal technology, USA); and actin (1 : 2000, cell signal technology, USA). The membrane was washed in TBST buffer and then incubated with the anti-mouse (1 : 1000, 7076, cell signal technology, USA) or anti-rabbit (1 : 1000, 7074, Cell Signaling Technology) secondary antibodies at 37°C for 2 hours. The protein was finally visualized through enhanced chemiluminescence detection method (SuperSignal West Dura Extended Duration Substrate, Thermo Fisher Scientific). The relative intensity of western blot was quantitatively evaluated using ImageJ software.

### 2.6. MTT Assay

Cells were seeded at a density of 5 × 10^3^/well in 96-well plates 24 hours after transfection. Following incubation at the indicated times (24 h and 48 h), 10 *μ*L MTT solution was added and the cells were incubated for another 4 hours at 37°C with 5% CO_2_. The absorbance was determined at 490 nm for each well using a microplate reader. Experiments were repeated in triplicate.

### 2.7. Apoptosis Assay

Cell apoptosis was quantitated using the annexin V-FITC/propidium iodide (PI) apoptosis kit (BD bioscience, USA). HK-2 cells were seeded into 6-well plates (5 × 10^5^ cells/well) at 37°C and subjected to cisplatin (5 *μ*g/mL) for 24 h. Cells were lifted from plates using trypsin (Gibco, USA). The treated cells were gently washed with phosphate-buffered saline (PBS) and resuspended in 500 *μ*L binding buffer. 5 *μ*L PI and annexin V-FITC were added to the mixture, and the cells were incubated for 30 min in the dark at room temperature. Finally, the apoptotic cells were examined using a flow cytometer. Annexin V positive/PI negative populations were considered cells in early apoptosis while annexin V positive/PI positive populations were considered cells in late apoptosis. Combination of the two populations was considered apoptotic. Experiments were repeated in triplicate.

### 2.8. Mitochondrial Membrane Potential

JC-1 staining method was used for the examination of the mitochondrial membrane potential (Δ*ψm*). Briefly, cells were washed with PBS buffer and then incubated with JC-1 fluorescent dyes (5 *μ*M) for 30 minutes in an incubator. The cells were washed with PBS buffer and applied to cytometer for analysis. The cut-off between green and red was accomplished by using CCCP treatment (50 *μ*M 15 minutes).

### 2.9. CellRox

The treated cells were collected and resuspended with serum-free medium. 5 *μ*M CellRox Deep Red reagent was added in the cells and the mixture was incubated for 30 minutes at 37°C. The supernatant was removed and PBS was used to wash the cells three times. The cells were then resuspended with PBS and analyzed using flow cytometry.

### 2.10. Mitotracker

The experimental steps were performed according to the instructions of Mitotracker Red Kit (Invitrogen, Carlsbad, CA, USA). A total of 100 nM mitotracker red solution was added to cells on a glass slide. The cells were incubated for 30 minutes at 37°C to stain the mitochondrial membrane. The staining solution was removed, and cells were observed using microscopy (BX53, Olympus, Tokyo, Japan). The relative mitochondrial length was quantitatively evaluated using ImageJ software.

### 2.11. Statistical Analysis

Data are expressed as the mean ± SD. SPSS 16.0 statistical software for Windows (SPSS, Chicago, IL, USA) was used for data analysis, and comparisons between subject groups were analyzed using Student's *t*-test. A value of *p* < 0.05 was considered significant.

## 3. Results

### 3.1. Sirt5 Expression Is Decreased in HK-2 Cells Treated with Cisplatin

**Figure 1:**
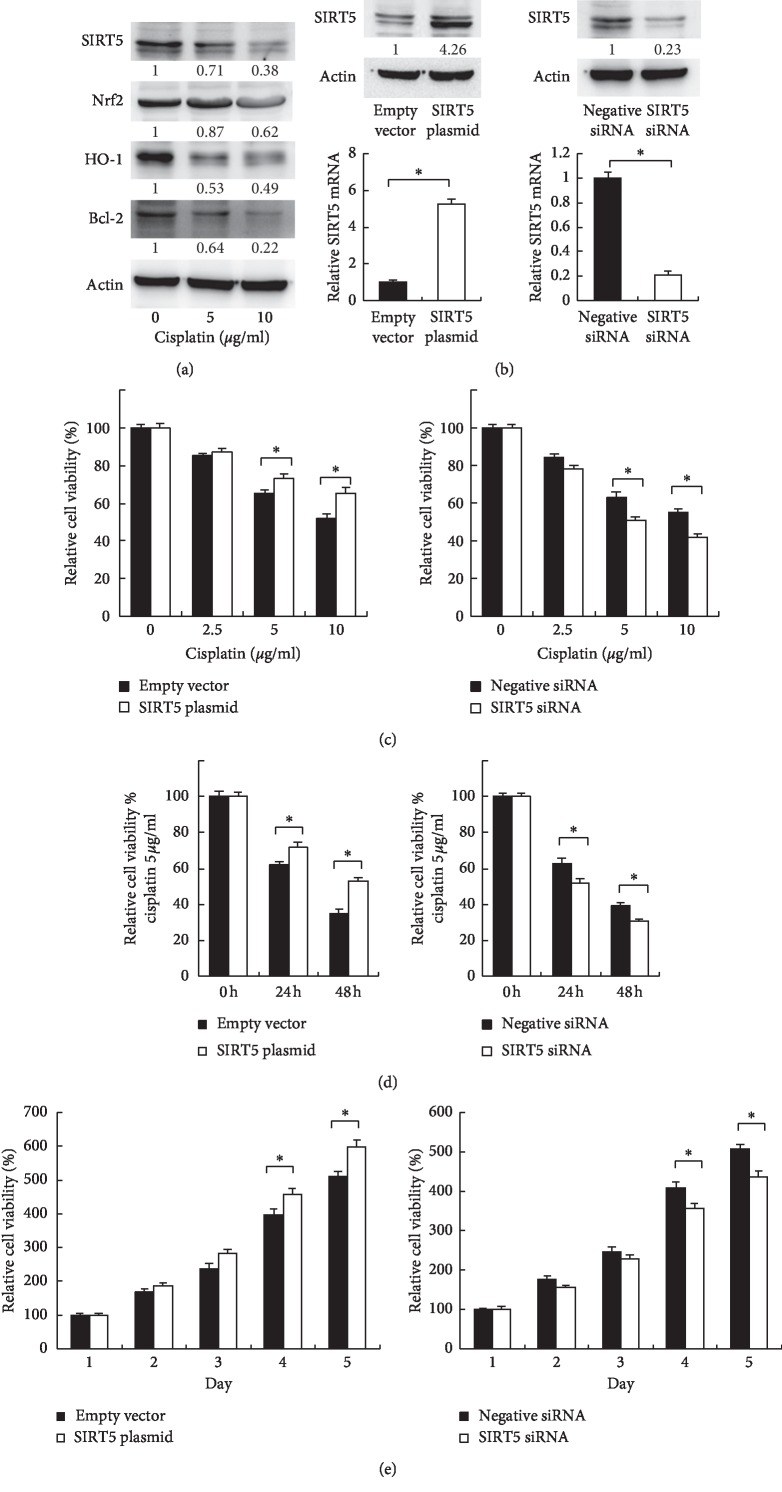
Sirt5 HK-2 metabolic activity treated with cisplatin. (a) Expression of Sirt5, Nrf2, HO-1, and Bcl-2 proteins in HK-2 cells treated with 0, 5, and 10 *μ*g/mL cisplatin for 24 hours. (b) Protein and mRNA expression of Sirt5 in HK-2 cells transfected with Sirt5 plasmid and siRNA. (c, d) MTT assay showed that Sirt5 overexpression increased metabolic activity while Sirt5 siRNA decreased metabolic activity when treated with different concentrations of cisplatin (0, 2.5, 5, and 10 *μ*g/mL) at different time points (24 and 48 hours). (e) Sirt5 overexpression increased HK2 growth rate. Sirt5 depletion decreased cell growth rate. ^*∗*^*p* < 0.05.

In this study, we used an ATCC cell line human kidney-2 (HK-2) which was derived from proximal tubule epithelium of the normal human kidney. We first examined the expression change of Sirt5 in HK-2 cells treated with cisplatin (5, 10 *μ*g/mL) for 24 hours. As shown in Figure 1(a), cisplatin treatment decreased protein expression of Sirt5 in a dose-dependent manner. In addition, the protein expressions of Nrf2, HO-1, and Bcl-2 were also decreased.

### 3.2. Sirt5 Increases Mitochondrial Metabolic Activity in HK-2 Cells Treated with Cisplatin

Next we checked if Sirt5 affected the viability of HK-2 cells treated with cisplatin. We overexpressed Sirt5 using plasmid transfection and depleted endogenous Sirt5 using siRNA knockdown. As shown in Figure 1(b), Western blots and real-time PCR analysis validated the efficiency of plasmid and siRNA transfection. MTT is an indicator of metabolic activity, specifically mitochondria. We then treated these cells with cisplatin and examined them using MTT assay. As shown in Figures 1(c) and 1(d), Sirt5 overexpression was able to maintain the metabolic activity while Sirt5 depletion decreased the metabolic activity when treated with different concentrations of cisplatin at different time points. We also examined cell proliferation rate after Sirt5 overexpression/depletion.  Figure 1(e) showed that Sirt5 overexpression increased HK2 growth rate, especially after 4 days of transfection. Sirt5 depletion decreased cell growth rate.

### 3.3. Sirt5 Regulates Apoptosis and Mitochondrial Function in HK-2 Cells

**Figure 2:**
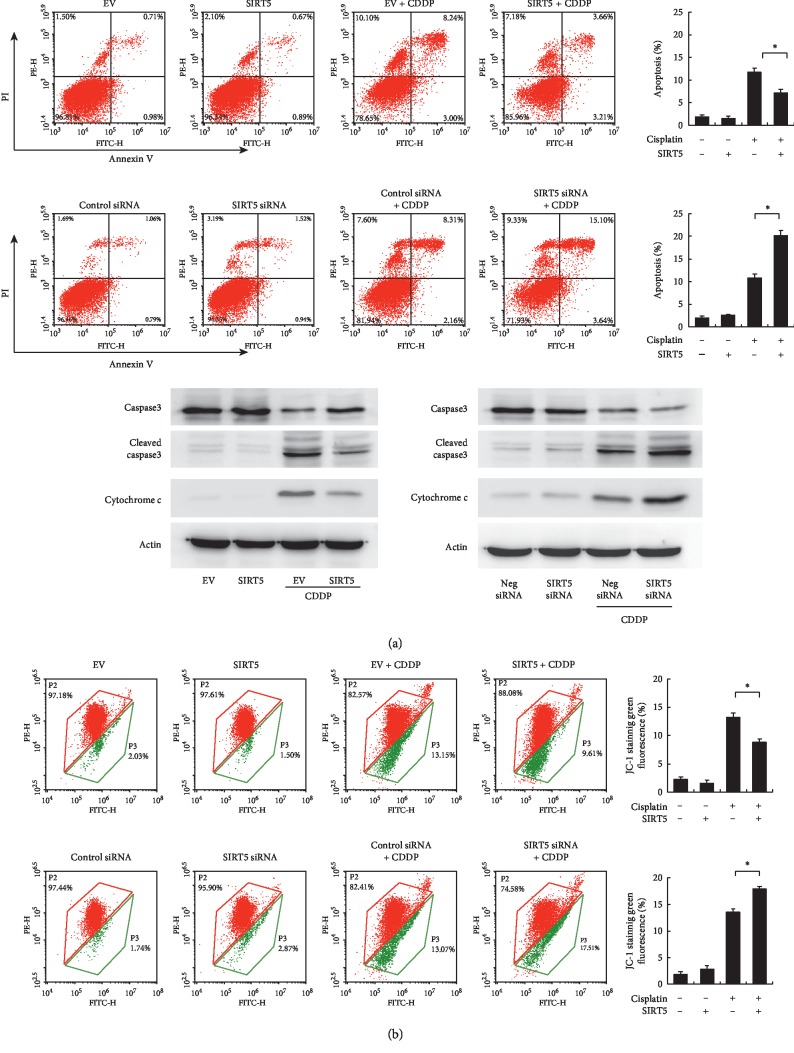
Sirt5 regulates apoptosis and mitochondrial membrane potential. (a) Annexin V/PI analysis of HK-2 cells with Sirt5 overexpression and knockdown. Sirt5 overexpression downregulates the rate of apoptosis in HK-2 cells treated with 5 *μ*g/mL cisplatin. Sirt5 siRNA knockdown upregulates the rate of apoptosis. Western blot showed that Sirt5 overexpression reduced the level of cleaved caspase 3 and cytochrome c in HK-2 cells treated with cisplatin. Sirt5 siRNA increased cleaved caspase 3 and cytochrome c protein. (b) JC-1 staining and flow cytometry showed that Sirt5 overexpression upregulated the mitochondrial membrane potential with decreased green fluorescence. Sirt5 siRNA knockdown decreased the mitochondrial membrane potential with increased green fluorescence after cisplatin treatment. ^*∗*^*p* < 0.05.

To determine the effect of Sirt5 on cisplatin-induced apoptosis, annexin V/PI staining was used. As shown in Figure 2(a), Sirt5 overexpression decreased the rate of apoptosis in HK-2 cells treated with 5 *μ*g/mL cisplatin. In contrast, Sirt5 depletion increased the rate of cisplatin-induced apoptosis. The change of apoptosis rate was not significant in cells without cisplatin treatment. These results indicated that Sirt5 reduces cisplatin sensitivity and conferred chemoresistance to HK-2 cells.

Because the sensitivity to chemotherapeutic drugs, including cisplatin, is related to the mitochondrial status, we examined whether Sirt5 affected the mitochondrial membrane potential (Δ*ψm*). JC-1 staining normally emits red fluorescence but turns into green when Δ*ψm* is decreased.

We used JC-1 staining and flow cytometry to monitor Δ*ψm* changes in HK-2 cells with or without cisplatin treatment. As shown in Figure 2(b), cisplatin treatment significantly decreased the Δ*ψm*. Sirt5 overexpression alleviated Δ*ψm* downregulation by cisplatin, while Sirt5 depletion showed the opposite effect.

To further validate the effect of Sirt5 on HK-2 cell apoptosis, we profiled several apoptosis related proteins including caspase 3, cleaved caspase 3, and cytochrome c. Sirt5 overexpression reduced the level of cleaved caspase 3 and increased cytochrome c expression. In contrast, Sirt5 depletion increased the level of cleaved caspase 3 and reduced cytochrome c.

**Figure 3:**
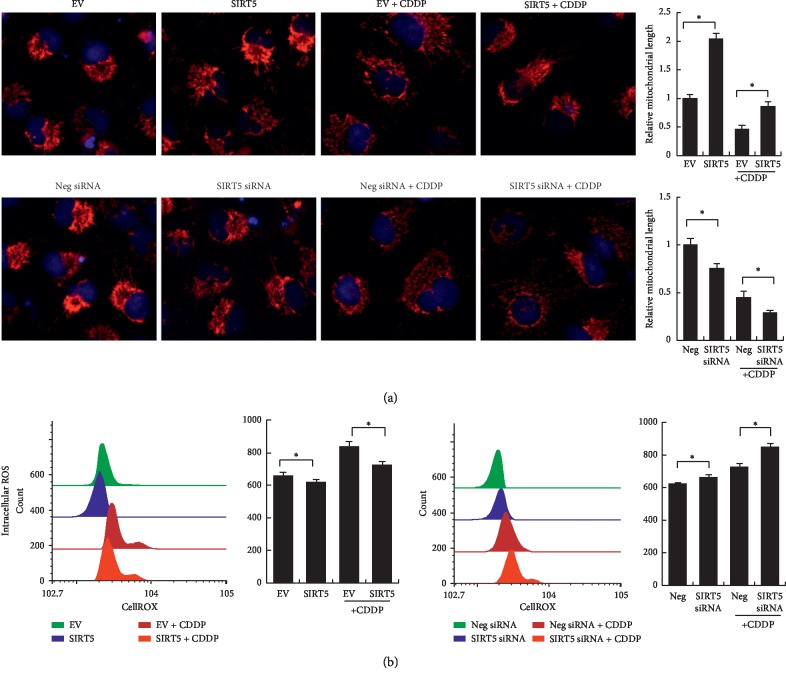
Sirt5 regulates mitochondrial morphology and ROS production. (a) Mitochondrial status (fusion/fission) was examined by Mitotracker staining. Cisplatin treatment induced mitochondrial fragmentation/fission. Sirt5 overexpression reduced mitochondrial fragmentation while Sirt5 depletion increased mitochondrial fragmentation. (b) Cellular ROS production was examined by CellRox staining. Cisplatin treatment elevated ROS level. Sirt5 overexpression reduced ROS production while Sirt5 depletion increased ROS level in HK-2 cells treated with cisplatin. The fluorescence intensity indicating ROS level was shown in the bar chart. ^*∗*^*p* < 0.05.

We also checked the change of mitochondrial status using Mitotracker staining, which showed intracellular mitochondrial levels and their fusion/fission status. As shown in Figure 3(a), cisplatin treatment reduced the total staining intensity and induced mitochondrial fragmentation. The relative mitochondrial length was quantitatively evaluated using ImageJ software. Sirt5 overexpression increased the total intensity and reduced mitochondrial fragmentation/fission. Sirt5 depletion showed the opposite effects.

### 3.4. Sirt5 Regulates ROS Production

To investigate the cisplatin-induced cellular reactive oxygen species (ROS) production, HK-2 cells were stained with CellRox Deep Red reagents and analyzed using flow cytometry. The results showed that cisplatin increased the fluorescence intensity. Sirt5 siRNA further enhanced the fluorescence intensity while Sirt5 overexpression reduced the fluorescence intensity, indicating that Sirt5 could reduce the intracellular ROS production.

### 3.5. Sirt5 Regulated Nrf-2/HO1 and Bcl-2 Signaling

**Figure 4:**
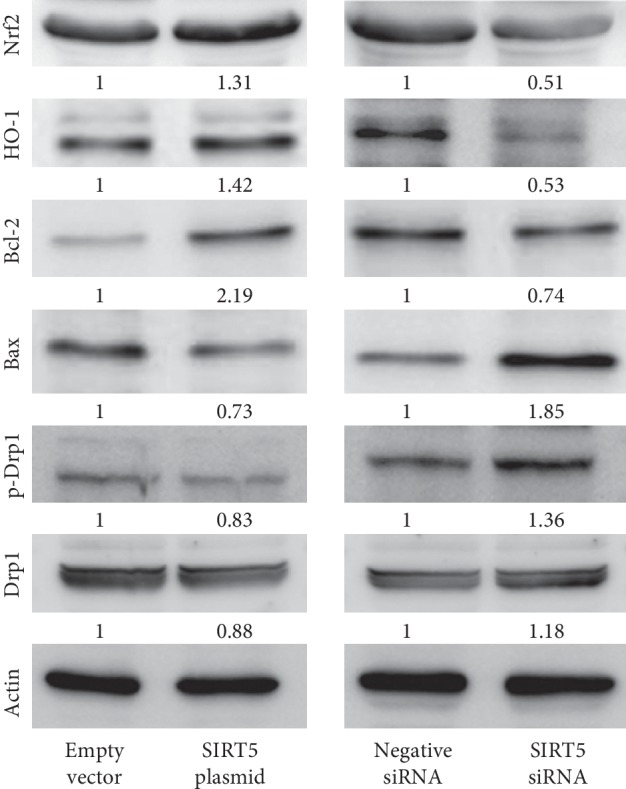
Sirt5 regulated Nrf-2/HO1 and Bcl-2. Western blot showed that Sirt5 overexpression upregulated Nrf2, HO-1, and Bcl-2 and downregulated Bax, p-Drp1, and Drp1 protein expression. Sirt5 siRNA downregulated Nrf2, HO-1, and Bcl-2 and upregulated Bax, p-Drp1, and Drp1 protein expression.

To elucidate the underlying mechanism of Sirt5 on apoptosis and mitochondrial function, we analyzed the expression of several related proteins. Nuclear factor erythroid 2-related factor 2 (Nrf2) is known as a redox-sensitive transcription factor and Heme oxygenase 1(HO-1) functions as its downstream effector. Nrf2 and HO-1 were reported to play critical roles in defense for oxidative stress. Bcl-2 has been regarded as an important regulator of the mitochondrial apoptosis pathway. Western blot analysis showed that Sirt5 overexpression increased expressions of Nrf2, HO-1, and Bcl-2 and decreased Bax protein expression. In contrast, Sirt5 depletion decreased Nrf2, HO-1, and Bcl-2 expressions and upregulated Bax expression (Figure 4). We also examined the expression of fusion/fission markers Drp1 and p-Drp1 (Ser616). Sirt5 overexpression inhibited Drp1 phosphorylation and downregulated total Drp1 expression while Sirt5 depletion showed the opposite effect.

### 3.6. Sirt5 Attenuates Apoptosis through Nrf2/HO-1 Signaling and Bcl-2

**Figure 5:**
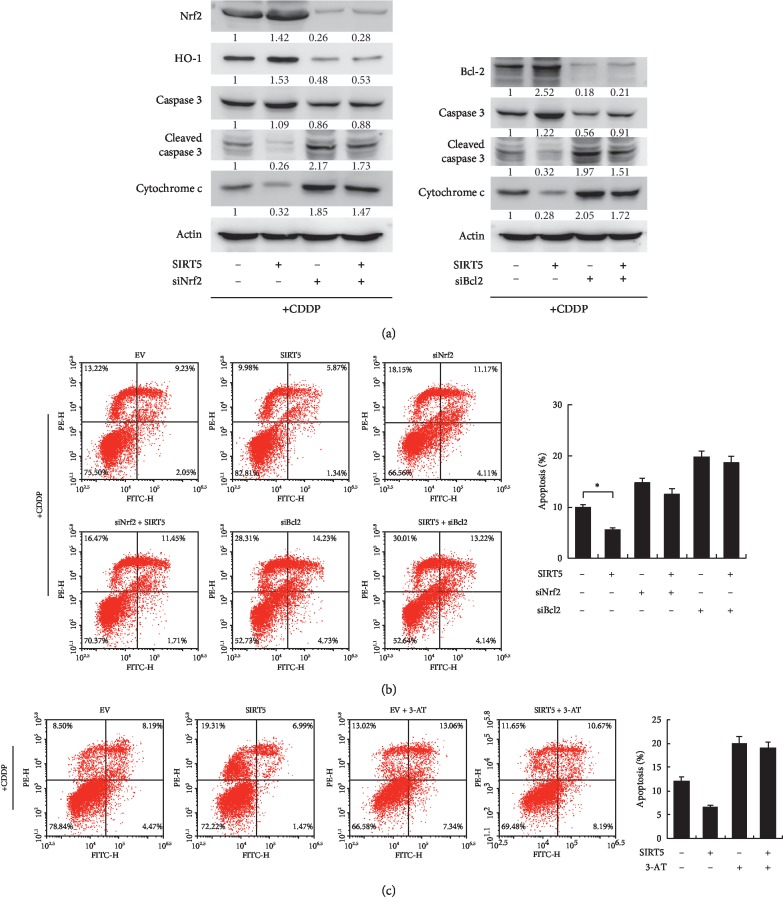
Sirt5 attenuates apoptosis through Nrf2/HO-1 and Bcl-2. (a) Nrf2 siRNA downregulates HO-1 protein expression. Both Nrf2 and Bcl-2 siRNA upregulated caspase 3 cleavage and cytochorme. (c) The protecting effect of Sirt5 on caspase cleavage and cytochrome c was reduced in cells with Nrf2 and Bcl-2 siRNA. (b) Nrf2 and Bcl-2 siRNA increased the rate of apoptosis. The protecting effect of Sirt5 on cisplatin-induced apoptosis was reduced in cells with Nrf2 and Bcl-2 siRNA. (c) Catalase inhibitor (3-amino-1,2,4-triazole, 3-AT) was used to treated HK2 cells (10 mM 3 hours). The rate of cisplatin-induced apoptosis was determined after Sirt5 overexpression. Sirt5 overexpression could not reduce apoptosis induced by cisplatin in cells treated with 3-AT. ^*∗*^*p* < 0.05.

Both Nrf2/HO-1 and Bcl-2 have been reported to be involved in mitochondrial stasis and apoptosis inhibition. To confirm whether Nrf2/HO-1 and Bcl-2 mediated the antiapoptotic role of Sirt5, we transfected siRNA targeting Nrf2 and Bcl-2 and examined the change of apoptosis and related proteins. As shown in Figure 5(a), Nrf2 siRNA decreased HO-1 protein expression. Sirt5 overexpression reduced caspase cleavage and cytochrome c release after cisplatin treatment. Both Nrf2 and Bcl-2 siRNA could partly abolish the protecting effect of Sirt5 on caspase cleavage and cytochrome c. In addition, Sirt5 overexpression reduced the rate of cisplatin-induced apoptosis while Nrf2 and Bcl-2 siRNA increased the rate of apoptosis (Figure 5(b)). The protecting effect of Sirt5 on cisplatin-induced apoptosis was reduced in cells with Nrf2 and Bcl-2 siRNA. We also investigated if cytoprotective effect of Sirt5 in cisplatin-treated cells was due to its antioxidant activity. We used a catalase inhibitor (3-amino-1,2,4-triazole, 3-AT), which inhibit catalase activity to protect the production of H_2_O_2_.  Figure 5(c) showed that after 3-AT treatment, Sirt5 could not reduce apoptosis induced by cisplatin, suggesting that the antioxidant activity may play an important role in the biological effects of Sirt5. Together, these results indicated that Sirt5 protected HK-2 from cisplatin-induced apoptosis partly through its regulation of Nrf2 and Bcl-2.

## 4. Discussion

Using an HK-2 cell model of cisplatin-induced nephropathy, we investigated whether Sirt5 could attenuate cisplatin-induced acute kidney injury (AKI). The pathogenic mechanisms responsible for cisplatin-induced nephrotoxicity were reported to be multifactorial including hypoxia microenvironment, oxidative stress, inflammatory stress, and apoptosis [22, 23]. Here, we showed that Sirt5 levels were decreased by cisplatin treatment. Restoration of Sirt5 maintained the metabolic activity and mitochondrial homeostasis and reduced apoptosis and ROS production. Furthermore, our mechanistic study indicated that the protecting role of Sirt5 may depend on its regulation on Nrf2/HO-1 and Bcl-2.

During cisplatin exposure, a large number of apoptotic cells were found to occur in the renal tissue. Consistent with a previous study [24], cisplatin treatment induced apoptosis of HK-2 cells. Sirt5 has been reported to have a strong desuccinylase activity [25]. The analyses of wild-type and Sirt5 knockout cells revealed several potential targets involving mitochondrial function including oxidative phosphorylation, tricarboxylic cycle, and amino acid metabolism [25, 26]. Thus, Sirt5 might be a key modulator of mitochondria homeostasis, which is essential during cell survival. The role of Sirt5 during apoptosis has not been reported in human kidney cells. Our data first demonstrated that Sirt5 overexpression partly reduced the toxic effect of cisplatin by downregulating the level of apoptosis, suggesting that Sirt5 played an important role in the survival of kidney cells under the cytotoxic pressure of cisplatin. It has been reported that resveratrol could prevent ROS-induced apoptosis through the activation of Sirt1 function [27, 28]. These data suggest that Sirt5 might be a potential target to protect kidney injury during chemotherapy. Finding Sirt5 activators may help to solve the disadvantage of cisplatin during clinical practice.

Because the mitochondria-dependent pathway plays a pivotal role in cisplatin-induced apoptosis [29–31], we examined the change of cytochrome c, caspase 3, and PARP cleavage. Cytochrome c binds to the inner mitochondrial membrane and released into the cytosol as the permeability of mitochondrial membrane increases, which could trigger cleavage of caspase/PARP and activation of apoptosis cascade. Our data confirmed that Sirt5 inhibited caspase 3 and PARP cleavage with reduced cytochrome c release, supporting its role in the inhibition of apoptosis progress.

Mitochondrial damage has been recognized as an important factor in the pathogenesis of cisplatin-mediated nephrotoxicity. Loss of mitochondrial membrane potential could increase mitochondrial membrane permeability, which increases cytochrome c release and triggers the mitochondria-dependent apoptosis pathway. Here, we checked the level of mitochondrial damage by examining mitochondrial membrane potential with JC-1 staining. We found that Sirt5 maintained a loss of mitochondrial membrane potential triggered by cisplatin treatment. We also used Mitotracker staining to examine mitochondrial dynamics. Cisplatin has been reported to induce mitochondrial fragmentation/fission and reduce total staining intensity, indicating mitochondrial damage and loss. Our data from HK-2 cells was consistent with previous reports, suggesting that directing targeting mitochondrial function, such as promoting mitochondrial biogenesis and mitophagy, could prevent kidney cell damage of AKI. In the Sirt family, Sirt1 activation has been reported to promote mitophagy and prevent ROS generation [32, 33]. Our data showed that Sirt5 reduced mitochondrial fragmentation/fission and intensity losses, suggesting a cytoprotective effect of Sirt5 by targeting mitochondria function in kidney cells.

Oxidative stress is considered an important issue in cisplatin-induced kidney damage. Reactive oxygen species (ROS) are able to interact and destroy the structure of different cellular components, such as DNA, proteins, and lipids. ROS production and accumulation lead to mitochondrial damage and apoptosis [34, 35]. Using CellRox staining, we showed that cisplatin-induced ROS was decreased after Sirt5 overexpression. Considering their connection, we adopted the catalase inhibitor to protect the production of ROS, and the cytoprotective effect of Sirt5 was significantly reduced with 3-AT treatment, suggesting that Sirt5 could reduce cisplatin-induced ROS, which maintained the membrane potential and inhibited cytochrome c release, eventually reducing apoptosis.

Mechanically, we identified Nrf2/HO-1 and Bcl-2 as a downstream target of Sirt5 in HK-2 cells. Nrf2 is a transcription factor which regulates the expression of several antioxidant genes and the activity of cytoprotective enzymes, contributing to the defense against oxidative stress. HO-1 is a powerful antioxidant that catalyzes the oxidation of the protein heme into antioxidant molecules, carbon monoxide, and biliverdin and improves cell survival. HO-1 acts downstream of Nrf2 [36–38], which has been shown to protect kidney cells against AKI induced by cisplatin [39]. Our data showed that Sirt5 positively regulated both Nrf2 and HO-1. In addition, Nrf2 knockdown reduced HO-1 expression and the protecting effect of Sirt5 against apoptosis. These results indicated that the protecting effect of Sirt5 may be partly due to its regulation of Nrf2/HO-1 signaling. Similarly, our data showed that Bcl-2 also mediated the protecting effect of Sirt5 against cisplatin in HK-2 cells. Bcl-2 is an anti-apoptotic protein which protects the cell from drug-induced apoptosis by reducing mitochondrial permeabilization and keeping its integrity [40, 41]. Overall, our findings linked the antiapoptotic role of Sirt5 to mitochondrial homeostasis, Nrf2/HO-1 and Bcl-2, which has not been previously reported in human kidney cells previously.

In conclusion, using HK-2 cells, we showed that Sirt5 attenuated cisplatin-induced acute kidney injury by activating Nrf2 and Bcl-2. We also identified novel roles for Sirt5 in kidney cells by linking antiapoptotic function with mitochondrial status and ROS production. Our data provides evidence that Sirt5 is crucial for metabolic homeostasis and cellular survival of kidney during cisplatin treatment. Further study is required to find out the activator of Sirt5.

## Data Availability

The present study does not contain data from published articles or database. The data used to support the findings of this study are available from the corresponding author upon request.
